# News consumption and green habits on the use of circular packaging in online shopping in Taiwan: An extension of the theory of planned behavior

**DOI:** 10.3389/fpsyg.2022.1025747

**Published:** 2022-10-10

**Authors:** Yi-Chih Lee

**Affiliations:** Department of International Business, Chien Hsin University of Science and Technology, Taoyuan, Taiwan

**Keywords:** news consumption, green habit, circular packaging, theory of planed behavior, COVID-19

## Abstract

The COVID-19 pandemic is far from over as outbreaks continue to spread around the world. The demand for packaging bags and cartons has also risen sharply in e-commerce shopping and takeaways because consumers have changed their shopping habits during the pandemic. The primary purpose of this study was to explore the factors prompting consumers to accept and use circular packaging when they shop online. From January to February 2022, a total of 373 online questionnaires were completed. The results showed that news consumption positively affected environmental attitudes, subjective norms, perceived behavioral control and circular packaging behavior intentions. Environmental attitudes, subjective norms, perceived behavioral control also affected the intention of circular packaging. Furthermore, news consumption influenced circular packaging behavioral intentions through environmental attitudes and green habits. Developing green habits in people’s daily lives will be of great help in guiding consumers to engage in other sustainable behaviors that are beneficial to life.

## Introduction

In addition to creating a massive crisis globally, the COVID-19 pandemic has also had a disastrous impact on efforts to achieve the Agenda 2030 for Sustainable Development. The Sustainable Development Goals Report 2021 pointed out that, despite the standstill of global human flows due to the pandemic, there are still climate and pollution crises. According to statistics, one million plastic beverage bottles are purchased every minute worldwide, and five trillion disposable plastic bags are discarded every year (United Nations Statistics Division, 2022). In addition, many problems with garbage have been caused by the pandemic, and the recycling business entirely stopped in some areas due to fear of spreading the virus (New Taipei City Government, 2021). With the worsening pandemic, people have utilized plastic gloves, plastic masks, and protective glasses in addition to face masks for personal protection and to avoid exposure. Disposable masks contain a large amount of polypropylene, which takes a long time to decompose, produces numerous toxins, and poses a potential threat to humans and the environment. At the same time, the large number of discarded masks has also increased ocean pollution (Awakening News Networks, 2020; World Health Organization, 2022). The pandemic has led many restaurants to revert to single-use cutlery, and they refuse to accept the environmentally beneficial containers brought in by consumers, again citing avoidance of exposure to accelerate disease transmission (Greenpeace, 2021c). The quarantine policies in various countries and the measures prohibiting people from going out non-essentially when the pandemic is severe have prompted many consumers to reduce physical face-to-face consumption and turn to shopping online and ordering food for delivery. These changes have led to an increase in household waste, including cardboard boxes and plastic bags for packaging as well as plastic products for delivery meals (Greenpeace, 2021a). The pandemic has intensified the consumption of disposable plastics, and the behavior of reducing plastics is decreasing.

Plastic is an inexpensive, lightweight, strong, and durable material especially suitable for packaging food and beverages. Plastic has become an indispensable material in people’s daily lives over the past decades. However, plastics persist for a long time after use and pose a significant threat to the environment and climate in the waste stage, such as contaminating soil and water sources (Gareiou et al., 2022). Half of the world’s plastic products are currently made for single-use, of which only 14% of plastic pack-aging is collected for recycling (World Economic Forum, 2022). As for the conservation of forest resources and the sustainable development of forests, trees can absorb and store carbon due to photo-synthesis, and forests help reduce the greenhouse effect, slowing global warming (Business Today, 2020a). The circular economy is a process that includes reducing, repurposing, remanufacturing, and recycling. Thus, reducing paper waste in the production process is essential (de Klerk et al., 2022). Using the most suitable carton to increase the volume ratio and reduce the amount of packaging material, using recycled paper materials, and testing different product combinations (Environmental Protection Administration, 2020), are all environmentally beneficial behaviors to save, recycle, and reuse resources.

Although measures to close the city or restrict movement during the COVID-19 pandemic were not implemented in Taiwan, various local counties and cities have temporarily encouraged the use of disposable tableware due to the fear of exposure. Policies banning in-restaurant dining have led to changes to takeaway or food delivery, with a sharp increase in recycled paper containers and plastic products. Domestic waste has increased by more than 20% after the outbreak of the pandemic, as compared with the amount of waste before the outbreak (Chinese Television System, 2021; Greenpeace, 2021b). Since the end of the COVID-19 pandemic is unknown, the most fundamental way to solve the plastic pollution crisis and reduce the impact of cutting down trees is to establish the environmental-protection habit of reuse (Greenpeace 2022d).

Herein, the current research on consumers’ green behaviors includes the theory of planned behavior to explore the factors affecting ecological behaviors (Wang and Wang, 2016; Hameed et al., 2019; Sharma and Foropon, 2019), the impact of green space on green behaviors (Kruize et al., 2019; Hamilton, 2020), and the impact of organizations on employees’ green behaviors (Francoeur et al., 2019; Li et al., 2021; Unsworth et al., 2021). Environmentally beneficial behaviors are a global trend, whereas the pandemic is an uncertain factor affecting green behaviors. The pandemic is not over yet, and it may have a recurrent effect on human behavior (Common Health Magazine, 2021). To reduce the chance of interpersonal contact, the consumption of single-use packaging materials in online shopping has been greatly increased, which highlights the dilemma between environmental protection and practical needs, and also has potential impacts on environmental pollution and human health. Even after the epidemic, personal selling may resume the use of environmentally friendly packaging, but packaging waste in online shopping may continue. Although there have been some studies on consumer behavioral changes in the use of plastic packaging during the pandemic (Liu et al., 2021; Leal Filho et al., 2022), there is still a lack of similar research on consumers’ willingness to accept circular packaging for online shopping. Therefore, the main purpose of this study was to explore which factors prompt consumers to accept and use circular packaging when they shopped online.

Therefore, the research questions are:

1. To explore the influence of people’s attitude, subjective norm, perceived behavioral control on their behavioral intentions to use circular packaging in online shopping.
2. To explore the influence of news consumption on attitudes, subjective norms, and perceived behavioral control to use circular packaging in online shopping.
3. To explore the mediating effect of habit in an extension model of the theory of planned behavior.

Consumers have the right not to participate in environmental protection. For any environmental protection plan, the key to success or failure lies on public engagement. Environmental protection is not a rigid demand of consumers, so they can achieve environmental protection goals only by encouraging and guiding them to take action continuously (Business Today, 2020b). Therefore, the most important contribution of this study was to identify key factors that will help consumers to adopt circular packaging for online shopping. The results of this study are expected to support both business opportunities and a healthy environment under the rapid development of the e-commerce market.

This paper first introduced the motivation, then conducted literature reviews and hypotheses, collected data to verify hypotheses, and finally put forward discussion and suggestions.

## Theoretical background and research hypotheses

### Environmentally friendly packaging

Packaging is the consumers’ subjective image of a commodity. Mueller Loose and Szolnoki (2012) pointed out that packaging design is critical to conveying the attributes of products to consumers (Mueller Loose and Szolnoki, 2012). There is currently no unified definition of circular packaging (Ketelsen et al., 2020), and different scholars have different definitions for circular packaging. Han et al. (2018) identified that sustainable or green packaging covers the three aspects of raw materials, production processes, and waste management (Han et al., 2018). The authors pro-posed using recycled materials and renewable energy as well as reducing the use of petroleum, which has an environmental impact. Environmentally friendly packaging should be produced in an energy-efficient manner, and the packaging should be as thin and light as possible. At the same time, at the end of the life cycle, the packaging should be biodegradable, reused, or recycled.

The Sustainable Packaging Coalition (SPC) under GreenBlue proposed five environmentally friendly product packaging methods, including taking a life cycle approach, considering the packaging and product relationship, choosing effective sustainability labeling and marketing, being creative, and talking with others. Packaging sustainability should be from the beginning of the design to the end of the life cycle and take into account such aspects as consumer usage and greenhouse gas emission factors. Packaging can prevent product damage and has communication benefits, so sustainable packaging for communicating with consumers is an important part of the development stage. Educating consumers regarding packaging issues will help manufacturers develop sustainable packaging materials. Furthermore, R&D personnel should strive to design more sustainable packaging materials and provide sustainable and practical dialogs in the product supply chain (GREENBIZ, 2013).

Presently, the common circular packaging materials are environmentally friendly cartons, packaging boxes, packaging bags, and packaging cases. Environmentally friendly cartons are made of recycled pulp and environmentally friendly water-based inks. Compared with plastic packaging, cartons decompose more easily and have a lower environmental burden. The environmentally friendly packing box is more environmentally friendly than plastic packaging and is usually used for food packaging and meal takeout or delivery. It avoids the risk of decomposition of plastic caused by hot food and thus prevents harm to the human body. Raw materials for circular packaging bags and environmental packaging boxes are diversified as renewable resources, bio-degradable materials, or paper bags are all material options for environmentally friendly packaging bags. Some environmentally friendly packaging bags have the functions of anti-fouling, impact resistance, waterproof, low carbon emissions, and can be reused to reduce environmental burden (PackAgeplus, 2021). In this study, circular packaging refers to packaging bags or boxes that consumers can reuse made from environmentally friendly packaging material.

Scholars have published related issues on reusable packaging and eco-packaging. Testa et al. (2020) pointed out the importance of seeking information in order to guide consumption choices that are more consistent with the circular economy. Geueke et al. (2018) focused on chemical safety aspects of recycled food packaging. Guillard et al. (2018) reviewed the major challenges that food packaging must tackle in the near future in order to enter the virtuous loop of circular bio-economy and they proposed some solutions. A study revealed that perceptions of eco-packaging directly influences consumer propensity to purchase and the positive relationship between perceptions of eco-packaging and purchases of eco-packaged goods is indirectly supported by an increase in an organization’s perceived brand equity and enhanced customer loyalty toward the organization (Zeng et al., 2017). Foschi and Bonoli (2019) described how European Commission has worked to regulate production and consumption patterns on plastic carrier bags and packaging thus facilitating the achievement of specific targets provided by the recent Directive. Ertz et al. (2017) used the theory of planned behavior to identify the mechanism about the consumption of reusable containers and showed that the context strongly impacts perceived behavioral control and motivations as well as attitudes. Besides, Attitude is a significantly stronger predictor of intentions for Westerners than Asians. Meherishi et al. (2019) presented a systematic literature review of studies to generate a greater understanding of the work done in the field of sustainable packaging in supply chain management (SPSCM). The review identified three main supply chain structures studied in SPSCM literature of which there has been an increased focus on fragmented portions and dyads of the supply chain with respect to packaging.

### News consumption and theory of planned behavior

Mass media is an important channel for the public to obtain information and knowledge of environmental issues. For environmental issues, according to the quantity of coverage theory, Mazur (2009) suggested that the quantity of media coverage on environmental issues, that is, the significance of their coverage, is even more important than the content, as most readers are more likely to be influenced by media messages than by the content (Mazur, 2009). If unpopular environmental issues can receive media attention, it will naturally affect the general public’s concern for environmental issues and gain public attention to environmental issues (Yang, 2020). Cox (2013) suggested that the media influences what issues the public consider. Furthermore, how the news is packaged also affects the perceptual construct of readers or viewers and evokes certain perceptions and values (Cox, 2013). In addition, the agenda-setting theory explains the effect of news media on the generation of public opinion. This theory claims that the quantity of the media coverage of an issue is proportional to the public’s perception of the importance of the issue; that is, the more the mass media reports on an issue, the deeper the psychological impression of the audience, and the higher the perceived importance of the issue (Dzwo, 2008). If the green packaging issue can receive media attention and there is a large quantity of media coverage, people’s consumption of the news on this issue will increase.

Media, social networks, or advertising can all affect personal attitudes. Online comments are a kind of information transmission, conveying the social impact of virtual communities. This kind of influence is similar to the impact of subjective norms on the individual in the theory of planned behavior. Jalilvand and Samiei (2012) pointed out that information communication can be a kind of transmission of information on other people’s past behavioral habits (Jalilvand and Samiei, 2012). It reflects how other people’s past experience of a certain behavior is the degree to which they perceive it to be easy or difficult to complete (Lee, 2021).

The theory of planned behavior suggests that behavioral intentions determine individual behaviors and that behavior intentions are determined by attitudes (whether I want it or not), subjective norms (other people’s opinions), and perceived behavioral control (whether I can do it or not; Ye, 2012). In theory, the attitude toward behavior refers to a person’s overall evaluation of a behavior, including behavioral beliefs and outcome evaluations. Meanwhile, behavior beliefs refer to the possible results of participating in a behavior, and outcome evaluation is the favored evaluation of the results of a behavior. Subjective norms include normative beliefs and motivation to comply. The former is the view of the important person of the individual regarding the individual’s participating behaviors, and the latter refers to whether individuals obey the opinions of others on their behaviors. Perceived behavioral control means that individuals evaluate the resources they have to judge whether they have enough control over a subsequent behavior (Wang et al., 2020). Ajzen (2005) pointed out that the measurement results of behavioral intentions can replace the performance of actual behaviors (Ajzen, 2005; Liao, 2022). At present, research on sustainable issues using TPB theory has achieved good results, including topics such as solving household food waste (La Barbera et al., 2022), the intention of food waste composting (Rahman et al., 2022), e-waste recycling behavior (Mohamad et al., 2022), and waste disposal *via* garbage exchange supermarkets (Lu et al., 2022).

Therefore, this study suggested that when a large number of people are exposed to online shopping news using circular packaging, it will have an impact on consumers’ behavioral attitudes, subjective norms, and perceived behavioral control. If an individual has a positive attitude toward a particular behavior, with the more subjective norms supporting the behavior and the stronger the perceived behavioral control, the higher the individual’s intention will be to engage in the behavior (Ye, 2012; Wang et al., 2020). Based on the above, the following hypotheses were proposed:

*H1*: News consumption has a positive impact on the behavioral intention to use circular packaging through attitude.

*H2*: News consumption has a positive impact on the behavioral intention to use circular packaging through subjective norms.

*H3*: News consumption has a positive impact on the behavioral intention to use circular packaging through perceived behavioral control.

### News consumption, attitude, habit, and behavioral intention

News consumption is information consumption, and information affects individuals’ attitudes (Chen and Tao, 2018). The theory of planned behavior (TPB; Ajzen, 1985) indicates that the performance of attitude can predict one’s likely behavior. When an individual’s attitude toward a behavior is more positive, the behavioral intention will be higher; conversely, if the attitude is more negative, the behavioral intention will be lower accordingly. Habits can be thought of as frequently repeating past behaviors; habits are, to some extent, automatic or non-subjective (Saba and Natale, 2008). Attitudes and habit processes are two important factors for coping with social problems, such as obesity or climate change, or adjusting one’s behavior in times of crisis during a pandemic. Attitude is a psychological tendency to evaluate the degree of liking or disliking a particular entity (Eagly and Chaiken, 1993). This study focused on attitude-to-behavior intentions, such as using recyclable bags or recyclable cartons to protect the environment. Therefore, here, attitudes can be understood as individuals’ evaluations of behaviors and their outcomes. Habits are memory-based tendencies that automatically respond to cues that lead to past behavioral performance. These tendencies are derived from memory cue-response associations in the context of a stable environment obtained by repeatedly responding to cues (Verplanken, 2018). This response may be an overt behavior or habitual thinking (Verplanken et al., 2007). Habits are often seen as social ills that need to be addressed; perhaps a healthier, safer, or more sustainable society may be created through a change in attitude. More importantly, however, habits play a major role in regulating desirable daily behaviors or consolidating long-term behavioral changes. Attitudes can be the starting point for habit formation (Verplanken and Orbell, 2022). In their investigation of the impact of different oils on food choices, Saba and Natale (2008) pointed out that oil consumption habits influenced oil purchase intentions (Saba and Natale, 2008); this finding clearly showed the influence of habits on behavioral intentions.

When people try something new, and it works, or people like it, the behavior may be repeated and eventually become a habit (Verplanken and Orbell, 2022). While habit formation may reinforce behavior changes, it also aligns behavioral change with the attitude that inspired it in the first place in a broader sense (Aarts and Dijksterhuis, 2000). If behaviors are something a person truly wishes to build, then a positive, strong, and stable attitude is a good place to start (Verplanken and Orbell, 2022). Furthermore, attitude is influenced by the amount of news information (Lee, 2021). Therefore, the following hypotheses were proposed by this study:

*H4*: News consumption has an impact on the behavioral intention to use circular packaging through attitude and habit.

## Data and methodology

### Research design

From January to the end of February 2022, the survey was conducted using convenience sampling. Convenience sampling is a common form of sampling found in population research. Convenience sampling is popular because it is not costly and simplistic. When used to generate a potential hypothesis or study objective, convenience sampling is useful (Stratton, 2021). We solicited voluntary participants in the study after stating the research purpose. We used questionnaire items developed by other scholars and tested the appropriateness of the questionnaire through reliability and validity. Due to covid-19, the questionnaire of this study was distributed using online questionnaires. This study involved a sample of 373 adults (aged 18 years or over) living in Taiwan. Demographic variables such as gender, age, marriage, and education were also collected in this study.

### Measures

The main variables in this study included news consumption, attitude, habits, subjective norms, perceived behavioral control and behavioral intention, which were measured in six main constructs, and a structured questionnaire was used as the measurement tool. The news consumption construct was mainly the circular packaging news consumption measure. The news consumption scale items were modified from those of Yoon et al. (2021) with a total of three items. The attitude construct mainly referred to an individual’s evaluation on the use of circular goods for environmental protection. The items of the attitude scale were modified from those of Hong and Fu (2012), with a total of six items. Habit referred to an individual’s behavior of environmental protection and product reuse in daily life. The items of the habit scale were modified from those of Untaru et al. (2020), and there were five items.

Subjective norms referred to individuals’ positive support or negative opposition to key reference groups for their use of circular packaging when shopping online and their willingness to comply with these norms. The items of the subjective norms scale were modified from those of Kamalanon et al. (2022), with a total of four items.

Perceived behavioral control referred to individuals’ ability to control the difficulty of choosing to use a recycling bag when the option of using a recycling bag is presented. The items of the perceived behavioral control scale were modified from those of Hong and Fu (2012) and Kamalanon et al. (2022), with a total of four items.

Behavioral intention referred to an individual’s willingness and the possibility to use circular packaging. The items of the behavioral intention scale were modified from those of Kamalanon et al. (2022), and there were four items in total. The 5-point Likert scale was adopted for measuring the scales, with five grades divided from strongly agree (5) to strongly disagree (1).

**Figure 1:**
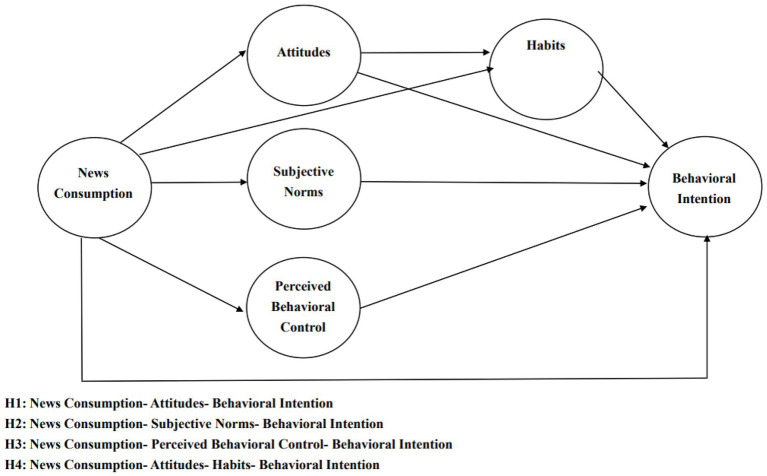
Shows the research model for the six constructs and their theoretical relationships.

To test the reliability and validity of the questionnaire, this study first conducted an analysis using Cronbach’s α. If it met 0.60, it showed good reliability (Kerlinger and Lee, 1999). Then, this study conducted a validity analysis using composite reliability (CR) and average variance extracted (AVE). For news consumption, Cronbach’s alpha was 0.948, CR was 0.966, and AVE was 0.906. For attitude, Cronbach’s alpha was 0.919, CR was 0.939, and AVE was 0.722. For habits, Cronbach’s alpha was 0.853, CR was 0.897, and AVE was 0.637. For subjective norms, Cronbach’s alpha was 0.873, CR was 0.913, and AVE was 0.723.For perceived behavioral control, Cronbach’s alpha was 0.867, CR was 0.910, and AVE was 0.718.For behavioral intention, Cronbach’s alpha was 0.873, CR was 0.929, and AVE was 0.766. This study achieved reliability coefficients for all constructs >0.7 and AVE > 0.5 (Hair et al., 2016). Generally speaking, in this study, the fitness of the measurement tool was positive (Table 1; Figure 1).

**Table 1:** The reliability, validity, and correlation of variables.

Variables	Cronbach’s α	CR	AVE	1	2	3	4	5	6
1. News Consumption	0.948	0.966	0.906	1					
2. Attitude	0.919	0.939	0.722	0.147^**^	1				
3. subjective norms	0.873	0.913	0.723	0.204^**^	0.596^**^	1			
4. perceived behavioral control	0.867	0.910	0.718	0.231^**^	0.695^**^	0.647^**^	1		
5. Habit	0.853	0.897	0.637	0.220^**^	0.482^**^	0.488^**^	0.648^**^	1	
6. Behavioral Intent	0.873	0.929	0.766	0.176^**^	0.661^**^	0.585^**^	0.749^**^	0.604^**^	1

** *p* < 0.01.

### Data analysis

This study used the Statistical Package for Social Sciences (version 21.0 IBM SPSS Inc., Chicago, IL, USA) to perform statistical analyses. Average mean, standard deviation, and percentage were used in the descriptive statistics. The statistical methods included the correlation analysis and the regression analysis to test the contribution and significance of the variables. This study also measured the Cronbach’s alpha, composite reliability (CR), and average variance extracted (AVE) of each variable to understand the reliability and validity of the scale. PROCESS Macro for SPSS was then used to verify the mediation effects. During all the testing of the PROCESS Models, this study used 10,000 bootstrap samples with 95 percent confidence intervals for the boot-strap analyses.

## Results

In the survey sample, the proportion of male and female respondents was nearly even (51.2 and 48.8%, respectively; Table 2). The age distribution was mostly 41–50 years old, accounting for 36.9%, followed by 51–60 years old, accounting for 24.6%. In terms of educational level, those with college education accounted for the highest proportion (46.1%), followed by those with a master’s degree or above (29.7%).

**Table 2:** The sample description (total *n* = 373).

Variables		Numbers	Percentage
Gender	Male	191	51.2%
	Female	182	48.8%
Age	<20	6	1.9%
	20–30	21	5.6%
	31–40	67	17.9%
	41–50	138	36.9%
	51–60	92	24.6%
	>60	49	13.1%
Marriage	Married	282	75.6%
	Single	87	23.3%
	Others	4	1.1%
Education	High school or below	90	24.2%
	University	172	46.1%
	Graduate school	111	29.7%

### News consumption-attitudes-behavioral intention

To verify whether the amount of news consumption had a significant impact on environmental attitude, a regression analysis was conducted with news consumption as the independent variable and attitude as the dependent variable. The results showed that news consumption had a significant positive impact on environmental attitude (beta = 0.147, *p* = 0.004); that is, the more consumers consumed circular packaging news, the more positive environmental attitude they would have. Secondly, the influence of news consumption on the use intention of circular packaging was tested. The results showed that news consumption had a significant positive impact on the use intention of circular packaging (beta = 0.176, *p* = 0.001); that is, the more consumers consumed circular packaging news, the higher their intention to use circular packaging. Furthermore, the effect of environmental attitude on the use intention of circular packaging was verified. The results showed that environmental attitude had a significant positive impact on the use intention of circular packaging (beta = 0.661, *p* < 0.001). In other words, when consumers had a positive environmental attitude, their intention to use circular packaging was higher. Finally, whether news consumption and environmental attitude had a significant impact on consumers’ intention to use circular packaging was tested. The results showed that the original news consumption (independent variable) had a significant relationship with the use intention of circular packaging (dependent variable). However, after the mediating variable (environmental attitude) was added, the relationship between the original independent variable and dependent variable weakened (beta decreased from 0.176 to 0.081, *p* = 0.040), and the mediating variable was significant (*p* < 0.001). Therefore, the environmental attitude had a partial mediating effect between news consumption and the intention to use circular packaging (Table 3). The results of the bootstrapping analyses conducted by PROCESS also supported this hypothesis. This study demonstrated that the indirect effect of news consumption on the intention to use circular packaging *via* environmental attitude was 0.0430, with a 95% confidence interval that did not contain zero (CI = [0.0146, 0.0721]).

**Table 3:** Analysis of news consumption and environmental attitudes toward the use of circular packaging.

Independent variable	Dependent variable	
	The intention to use circular packaging	
	Model I	Model II
News consumption	0.176**	0.081*
Environmental attitude		0.650***
*F* value	11.851***	147.588***
*R* ^2^	0.031	0.444

* *p* < 0.05;

** *p* < 0.01;

*** *p* < 0.001.

### News consumption-subjective norms − behavioral intention

To verify whether the amount of news consumption had a significant impact on subjective norms, a regression analysis was conducted with news consumption as the independent variable and subjective norms as the dependent variable. The results showed that news consumption had a significant positive impact on subjective norms (beta = 0.204, *p* < 0.001); that is, the more consumers consumed circular packaging news, the more positive subjective norms they would have. Secondly, the influence of news consumption on the use intention of circular packaging was tested. The results showed that news consumption had a significant positive impact on the use intention of circular packaging (beta = 0.176, *p* = 0.001); that is, the more consumers consumed circular packaging news, the higher their intention to use circular packaging. Furthermore, the effect of subjective norms on the use intention of circular packaging was verified. The results showed that subjective norms had a significant positive impact on the use intention of circular packaging (beta = 0.585, *p* < 0.001). In other words, when consumers had a positive subjective norm, their intention to use circular packaging was higher. Finally, whether news consumption and subjective norms had a significant impact on consumers’ intention to use circular packaging was tested. The results showed that the original news consumption (independent variable) had a significant relationship with the use intention of circular packaging (dependent variable). However, after the mediating variable (subjective norm) was added, the relationship between the original independent variable and dependent variable weakened (beta decreased from 0.176 to 0.059, *p* = 0.169), and the mediating variable was significant (*p* < 0.001). Therefore, the subjective norm had a perfect mediating effect between news consumption and the intention to use circular packaging (Table 4). The results of the bootstrapping analyses conducted by PROCESS also supported this hypothesis. This study demonstrated that the indirect effect of news consumption on the intention to use circular packaging *via* the subjective norm was 0.1167, with a 95% confidence interval that did not contain zero (CI = [0.0571, 0.1752]).

**Table 4:** Analysis of news consumption and the subjective norm toward the use of circular packaging.

Independent variable	Dependent variable	
	The intention to use circular packaging	
	Model I	Model II
News consumption	0.176**	0.059
Subjective norm		0.573***
*F* value	11.851***	177.513***
*R* ^2^	0.031	0.345

* *p* < 0.05;

** *p* < 0.01;

*** *p* < 0.001.

### News consumption-perceived behavioral control − behavioral intention

To verify whether the amount of news consumption had a significant impact on perceived behavioral control, a regression analysis was conducted with news consumption as the independent variable and perceived behavioral control as the dependent variable. The results showed that news consumption had a significant positive impact on perceived behavioral control (beta = 0.231, *p* < 0.001); that is, the more consumers consumed circular packaging news, the more positive perceived behavioral control they would have. Secondly, the influence of news consumption on the use intention of circular packaging was tested. The results showed that news consumption had a significant positive impact on the use intention of circular packaging (beta = 0.176, *p* = 0.001); that is, the more consumers consumed circular packaging news, the higher their intention to use circular packaging. Furthermore, the effect of perceived behavioral control on the use intention of circular packaging was verified. The results showed that perceived behavioral control had a significant positive impact on the use intention of circular packaging (beta = 0.749, *p* < 0.001). In other words, when consumers had a positive perceived behavioral control, their intention to use circular packaging was higher. Finally, whether news consumption and perceived behavioral control had a significant impact on consumers’ intention to use circular packaging was tested. The results showed that the original news consumption (independent variable) had a significant relationship with the use intention of circular packaging (dependent variable). However, after the mediating variable (perceived behavioral control) was added, the relationship between the original independent variable and dependent variable weakened (beta decreased from 0.176 to 0.003, *p* = 0.922), and the mediating variable was significant (*p* < 0.001). Therefore, the perceived behavioral control had a perfect mediating effect between news consumption and the intention to use circular packaging (Table 5). The results of the bootstrapping analyses conducted by PROCESS also supported this hypothesis. This study demonstrated that the indirect effect of news consumption on the intention to use circular packaging *via* the subjective norm was 0.1725, with a 95% confidence interval that did not contain zero (CI = [0.1018, 0.2440]).

**Table 5:** Analysis of news consumption and the perceived behavioral control toward the use of circular packaging.

Independent variable	Dependent variable	
	The intention to use circular packaging	
	Model I	Model II
News consumption	0.176**	0.003
Perceived behavioral control		0.748***
*F* value	11.851***	446.134***
*R* ^2^	0.031	0.561

* *p* < 0.05;

** *p* < 0.01;

*** *p* < 0.001.

**Figure 2:**
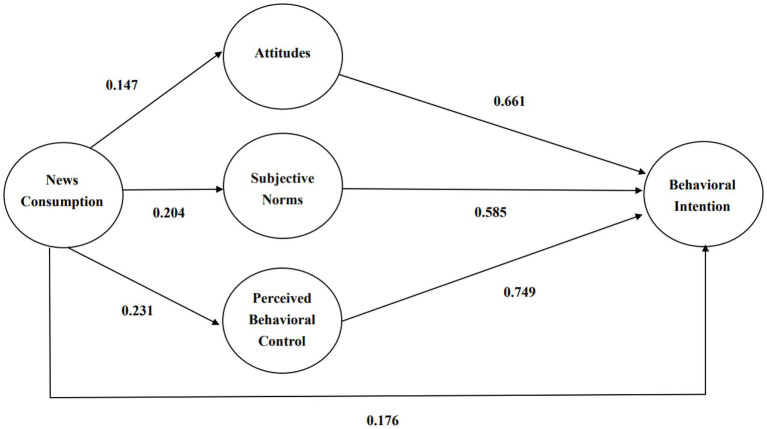
The overall model of multiple regression analysis.

The overall model of multiple regression analysis is as Figure 2.

### News consumption-attitudes- habits-behavioral intention (serial multiple mediation model)

**Figure 3:**
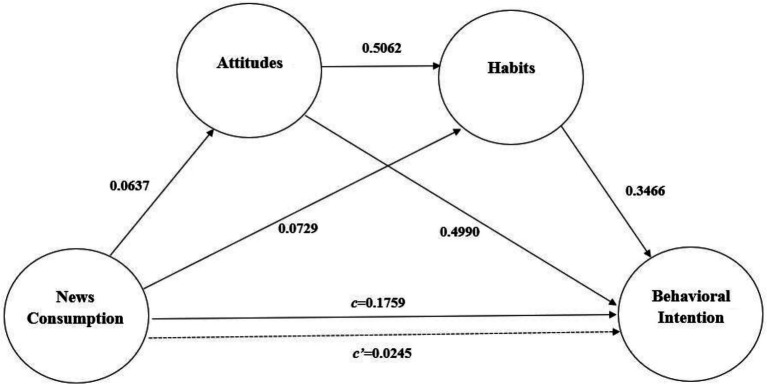
Path analysis of news consumption, attitudes, habits, and behavioral intention.

As can be seen in Figure 3, total effect (c = 0.1759, SE = 0.0230, *t* = 3.4426, *p* = 0.0006) of News consumption on behavioral intention was at a significant level. In addition, the direct effects of news consumption on attitudes (B = 0.0637, SE = 0.0223, *t* = 2.8587, *p* = 0.0045) and habits (B = 0.0729, SE = 0.0217, *t* = 3.3680, *p* = 0.0008) were at significant levels. The direct effect of attitudes as the first mediating variable on the second mediating variable of habits (B = 0.5062, SE = 0.0499, *t* = 10.1448, *p* < 0 0.001) is on significant level. A review of the direct effects of mediating variables on behavioral intention showed that the effects of attitudes (B = 0.4990, SE = 0.0417, *t* = 11.9636, *p* < 0.001) and habits (B = 0.3466, SE = 0.0384, *t* = 9.0148, *p* < 0.001) were at significant levels. When news consumption and all other mediating variables were simultaneously entered into the equation, the relationship between news consumption and behavioral intention, in relation to direct effect, was not at a significant level (c’ = 0.0245, SE = 0.0163, *t* = 0.6792, *p* > 0.05). Based on this result, the mediating variables were observed to mediate between news consumption and behavioral intention. In addition, the model overall was seen to be at a significant level (*F* = 146.8257, p < 0.001) and explained 54.42% of the total variance in behavioral intention.

The comparison of indirect effects and specific effects of news consumption on behavioral intention levels through attitudes and habits is included in Table 6. Statistical significance of the indirect effects within the tested model in the current research was examined over 10,000 bootstrap samples. Estimates were taken at a 95% confidence interval, and the results are presented in Table 6. The total indirect effect of news consumption through attitudes and habits on behavioral intention is statistically significant (estimate =0.0682; 95% CI [0.0363, 0.1006]). Within the tested model, when considering the mediating variables separately and together in relation to the mediating indirect effects of news consumption on behavioral intention, single mediation of attitudes (estimate = 0.0318; 95% CI [0.0107, 0.0538]), serial-multiple mediation of attitudes and habits (estimate = 0.0112; 95% CI [0.0037, 0.0195]), and single mediation of habits (estimate = 0.0253; 95% CI [0.0108, 0.0419]) were found statistically significant. Contrasting findings presented in pairs were included in the current research in order to determine whether specific indirect effects of mediating variables were stronger than others. Only one statistically significant contrast not within the zero-point estimate based on the 95% confidence interval has been presented in Table 6. Based on the contrasting pair of specific indirect effects, the variable of attitudes was observed to have stronger mediation than the serial-multiple mediation of attitudes and habits.

**Table 6:** Comparison of the indirect effects of news consumption on behavioral intention through attitudes and habits and its specific indirect effects.

	Coefficients		95% Confidence interval	
Effects	Estimate	SE	Lower	Upper
Total Indirect Effects	0.0682	0.0164	0.0363	0.1006
News consumption- > attitudes- > behavioral intention (Model 1)	0.0318	0.0111	0.0107	0.0538
News consumption- > habits- > behavioral intention (Model 2)	0.0253	0.0079	0.0108	0.0419
News consumption- > attitudes- > habits- > behavioral intention (Model 3)	0.0112	0.0040	0.0037	0.0195
Contrasts				
Model 1 versus Model 2	0.0065	0.0139	−0.0209	0.0333
Model 1 versus Model 3	0.0206	0.0083	0.0062	0.0382
Model 2 versus Model 3	0.0141	0.0085	−0.0018	0.0319

## Discussion and implications

### Discussion

As the COVID-19 pandemic continues, the global home economy is developing rapidly, and e-commerce turnover continues to rise (Lin, 2021). These events have resulted in the use of a large amount of packaging materials. The piles of cartons and bubble wrap materials indicate that consumption has caused an increased burden on garbage disposal and a crisis of environmental protection. With the large-scale consumption of online shopping and takeaway food, it has become a priority to promote green pack-aging to make consumers identify with green packaging and to actively use it to reduce the garbage threatening the environment. The findings of this research identified that the reinforcement of media news could deepen people’s environmental protection attitudes, subjective norms, perceived behavioral control and develop green habits in people’s daily lives, and be of great help in guiding consumers to engage in other sustainable behaviors that are beneficial to life.

The theoretical contribution of this study is that the model, built solely on the basis of TPB, proved predictive of the intention to use circular packaging for online shopping; importantly, it also added two specific factors, news consumption and green habits, which have been shown to influence usage intention. This research has extended the application of TPB theory.

### Theoretical implications

First, this study found that the consumption of circular packaging news positively impacted people’s environmental protection attitude and the use of circular packaging, which agrees with previous research (Lee, 2021; Lee et al., 2021). At the same time, people’s environmental attitudes played a mediating role. The large-scale involvement of green packaging media news could enhance consumers’ attitudes toward environmental protection and further strengthen the public’s willingness to use circular packaging. Therefore, to change the behavior of the public to use circular packaging, strengthening the media publicity of green packaging is the first and foremost task. Furthermore, although news related to recycling packaging could affect subjective norms and perceived behavioral control, the behavioral intention for consumers’ use of recycling packaging was mainly affected by subjective norms and perceived behavioral control, which agreed with previous research (Rahman et al., 2022).

In addition, our study also found that news consumption was positively associated with people’s environmental attitudes, then with their daily environmental habits, which in turn positively contributed to behavioral intention toward using circular packaging. The environmental attitude and habit played mediating roles. If the public was more exposed to recycling bag information, people had a more positive attitude toward environmental protection, as well as they would also engage in environmental protection habits in their daily life, and their intention to have new environmentally friendly behaviors (the use of circular packaging) would also increase. This is similar to the findings of Untaru et al. (2020). Stern (2000) identified that different types of environmental protection behaviors are affected by attitude factors, personal abilities, environmental factors, habits, and practices, with different degrees of influence (Ketelsen et al., 2020; Mohamad et al., 2022). Thus, the development of public environmental protection attitudes and the establishment of environmental protection habits are important factors for promoting new environmental issues. When the public already has an attitude and habit of environmental protection, it means that they agree with the awareness of a healthy environment, and they are more willing to participate in related sustainable actions that are beneficial to environmental protection.

### Managerial implications

This study proposed several management implications. First, it is important to utilize multiple media sources, including traditional electronic media (e.g., TV, newspapers, or periodicals) and online social media (e.g., FB, Instagram, TikTok, Podcast, YouTube, and other channels). Starting discussions with green packaging videos will link circular packaging, citizen awareness, a favorable environment, and other altruistic behaviors to deepen public environmental protection attitudes, thereby promoting the public’s willingness to use circular packaging. Second, the influence of important reference groups is an important factor in changing consumers’ willingness to use recycled packaging when shopping online. Therefore, educational institutions can promote the use of recycled packaging in online shopping on campus, provide small activities to increase participant motivation, or ally with eco-friendly YouTubers to make an impact on the public. Third, perceived behavioral control is also one of the driving forces that influences the use of circular packaging. It is recommended to strengthen the convenience of recycling packaging, such as reminding consumers to use recycled packaging bags at the last step of shopping or combining e-commerce goods distribution centers, schools, supermarkets, public agencies, stores, and other places where the public often go, to strengthen recycling. The more convenient the process is provided by stores, the more willing consumers will be to join the use of recycled packaging. Finally, Circular packaging can be promoted among people with environmental attitudes and habits first. By starting with the consumption points that these groups are often exposed to, the concept of circular packaging will be gradually promoted in people’s daily life. These consumption points include setting up a green e-commerce platform to guide consumers to use green packaging when purchasing or introducing incentives on the platform for consumers who use green packaging to accumulate feedback points or discounts.

### Limitation and future research

Based on the suggestions of Ketelsen et al. (2020), this study explored consumers’ responses to specific packaging (circular packaging for online shopping; rather than general eco-friendly packaging) in order to gain a deeper understanding of consumers’ intention to accept specific eco-friendly solutions. This study only discussed external information, TPB and habits. Many factors affect consumers’ choice of environmental protection behavior. It is suggested that subsequent research could divide consumers into groups according to their environmental protection habits for analysis and promote beneficial programs to consumers with different degrees of environmental protection use. In addition, different countries have different practices for developing green packaging for e-commerce, and the degree of popularization also varies. It is recommended that subsequent research includes a detailed analysis of the development policies of different countries to provide peer bench-marking. Third, most studies focus on demographic variables for comparison, with less exploration of the applicability of circular packaging for different cultures. It is suggested that follow-up researchers try to find solutions to the barriers to promoting green packaging from the cultural level.

### Conclusion

The COVID-19 epidemic and the development of e-commerce have increased human reliance on single-use plastics, but we must continue to take sustainable actions. The findings indicated that the behavioral intention to use circular packaging in e-commerce shopping is affected by news consumption, attitudes, habits, subjective norms and perceived behavioral control, so starting from these factors to change people’s behavior will greatly improve the reuse of environmentally friendly packaging.

## Data availability statement

The raw data supporting the conclusions of this article will be made available by the author, without undue reservation.

## Author contributions

The author confirms being the sole contributor of this work and has approved it for publication.

## Conflict of interest

The author declares that the research was conducted in the absence of any commercial or financial relationships that could be construed as a potential conflict of interest.

## Publisher’s note

All claims expressed in this article are solely those of the authors and do not necessarily represent those of their affiliated organizations, or those of the publisher, the editors and the reviewers. Any product that may be evaluated in this article, or claim that may be made by its manufacturer, is not guaranteed or endorsed by the publisher.

## Appendix

**Table:**

Variables	Items
News Consumption	I have read news or information about the circular packaging in online shopping on social media (e.g., Facebook, Twitter, Instagram) I have read news or information about the circular packaging in online shopping *via* instant messaging platform (e.g., text messages, Line) I have read news about the circular packaging in online shopping (e.g., newspaper articles, magazine, Google News)
Attitude	Using the circular packaging in online shopping can reduce negative environmental impacts Using the circular packaging in online shopping can reduce costs Using secondary sources can mitigate negative environmental impacts Let many people know and implement the circular packaging in online shopping Reducing the waste of resources can indirectly reduce the negative impact on the environment Using the circular packaging in online shopping can make our lives more livable
Subjective norms	Mass media in the publicity will cause me to use the recycling bag/box in online shopping The work environment will make me use recycle bags/boxes in online shopping My parents’ request will let me use recycled bags/boxes in online shopping People around me influence my use of recycled bags/boxes in online shopping
Perceived behavioral control	In daily life, I will reuse resources I can protect the environment by using recycled bags/boxes in online shopping Every consumer can have a positive impact on society by purchasing products sold by socially responsible companies Everyone has an impact on pollution and natural resources, so what I do can make a difference
Habit	I replace plastic bags with reusable bags I bring my own food storage containers to store meat, fish, etc., and even cooked food I bring my own water bottle to reduce the need for bottled water I bring my own reusable food ware every day I use rags instead of disposable kitchen towels
Behavioral Intent	I am willing to use recycled bags/boxes in online shopping If the price is not different from other prices, I will use recycle bags/boxes while online shopping If the quality is no different from others, I will use recycled bags/boxes *while online shopping* I will consider switching to use other packaging products due to environmental factors.
